# 

**Published:** 1861-12

**Authors:** 


### BOOKS RECEIVED.

*Lectures on Materia Medica and Therapeutics, delivered in the College of Physicians and Surgeons of the University of New York. By* JOHN B. BECK, M. D., *late Professor of Materia Medica and Medical Jurisprudence. Prepared for the Press by his friend,* C. R. GILMAN, M. D., *Professor of Obstetrics, etc., in the College of Physicians and Surgeons, N. Y. Third Edition. New York:* SAMUEL S. & WILLIAM WOOD, 389 *Broadway,* 1861.

We shall take pleasure in noticing this book as soon as space will allow. We can now only say, it should receive the attention of not only physicians, but of medical students who desire to have the most recent and correct treatise upon Materia Medica. We have received this book from THEODORE BUTLER, No. 159 Main street, Buffalo, where those desiring to purchase can be supplied.

*Lectures on the Diseases of Women, by* CHARLES WEST, M. D., *Fellow of the Royal College of Physicians; Examiner in Midwifery at the Royal College of Surgeons of England; Physician Accoucher to St. Bartholomew's Hospital; and Physician to the Hospital for sick Children; author of "Lectures on the Diseases of Infancy and Childhood." Second American, from the Second London Edition. Philadelphia:* BLANCHARD & LEA, 1861.

